# Second-order robust finite difference method for singularly perturbed Burgers' equation

**DOI:** 10.1016/j.heliyon.2022.e09579

**Published:** 2022-05-31

**Authors:** Masho Jima Kabeto, Gemechis File Duressa

**Affiliations:** Department of Mathematics, Jimma University, Jimma, Ethiopia

**Keywords:** Singularly perturbed, Burgers' equation, Accurate solution

## Abstract

In this paper, a second-order robust method for solving singularly perturbed Burgers' equation were presented. To find the numerical approximation, we apply the quasilinearization technique before formulation of the scheme. The obtained experimental results show that the presented method has better numerical accuracy and convergence as compared to some existing methods in the literature. Thus, the present method provides an accurate solution and efficient to solve the singularly perturbed Burgers' equation.

## 1 Introduction

We considered the singularly perturbed Burgers' equation:

(1.1) $$\frac{\partial u(x,t)}{\partial t}-\varepsilon \frac{\partial^{2}u(x,t)}{\partial x^{2}}+u(x,t)\frac{\partial u(x,t)}{\partial x}=0,\forall (x,t)\in \Omega ,$$

subjected to the initial and boundary conditions

(1.2) $$\left\{ \begin{array}{cc} & u(x,0)=s(x),\;\;\;0\leq \;x\;\leq 1,\\ & u(0,t)=q_{0}(t),\;\;u(1,t)=q_{1}(t),\;\;\;0<\;t\;\leq T, \end{array}\right.$$

where $\Omega =\Omega_{x}\times \Omega_{t}=(0,1)\times (0,T]$ is domain, for *T* is positive constant. The small positive constant *ε*, is called the perturbation parameter. Assume that the consistence conditions are satisfied $s(0)=s(1)=q_{0}(0)=q_{1}(0)$, to admits a unique solution u(x,t).

Numerical methods for solving singularly perturbed parabolic differential equation depend upon parameters, which causes the solution to fluctuate fast in some parts of the domain and varies slowly in some other parts. Hence, several numerical methods have been developed by different scholars to solve such equations, and the need to find numerical methods for approximating its solution is valuable. Singularly perturbed parabolic problems arise in numerous branches of science and engineering. The famous examples are the Navier-Stokes equation with large Reynolds number in fluid dynamics and the convective heat transport problems with large Peclet number. Also, these problems have different properties and modeled problems depending on the dimensions, number of parameters involved, natures of the models, etc. For instance, some of them are singularly perturbed Burgers' equations, Burger-Huxley equations, Burger-Fisher equations, and so on, see the reference in [1], [2], [3], [4], [5], [6], [7], [8], [9].

Singularly perturbed Burgers' equation first introduced by Bateman in 1915 [9] is used in the modeling of the motion of the viscous fluid. Later in 1948, Burgers' in his work [10] formulated this equation from the Navier-Stokes equation that can model the theory of turbulence flow designated by the interaction of two contradictory effects of convection and diffusion. This equation occurs in various areas of applied mathematics such as fluid mechanics, turbulence flow, reservoir simulation, continuous stochastic processes, and shock waves [10], [11], [12], [13], [14].

Several classical methods have been developed for solving singularly perturbed Burgers' equation. Some of these methods can be categorized as finite difference [5], [11], [14], finite element [15], [22] and finite volume methods [16]. Most of these methods are in difficulty to solve singularly perturbed Burgers' equations when the perturbation parameter approaches zero unless a very fine mesh is considered, which ill-advisedly rises the computational complexity. Thus, this motivates researcher to formulate a higher-order numerical method in order to obtain a more accurate solution for singularly perturbed problems.

## 2 Statement of the problem

For the considered singularly perturbed nonlinear parabolic partial differential equation given in Eq. (1.1)–(1.2), evidently, different scholars [1], [2], [6], [9], [10], [12], [20], devoted to obtain more accurate solutions by using different techniques. Moreover, recently [1] constructed a robust adaptive grid method and [12] developed the implicit Finite Difference for the problem under Eq. (1.1). Though, the obtained solution desires enhancement which directs that still such kind of problems requests developments to alternative methods to yield a more accurate numerical solution. To achieve this purpose, the nonlinear terms are linearized by means of quasi-linearization technique with reasonable initial guess and then second-order robust method is formulated. Further, the real-time application of the modeled problem of Eq. (1.1), its continuous and discrete properties such as existence, stability and boundness of the solution are discussed in the literature [1], [12], [14], [23].

## 3 Formulation of the numerical scheme

To linearize the linear term, from separation of variables method to solve the one dimensional homogenous heat equation, let us consider the reasonable initial approximation:

(3.1) $$u^{\left(0\right)}(x,t)=s(x)exp(-\pi^{2}t).$$

Then, applying the quasilinearization procedures at the first iteration on $u(x,t)\frac{\partial u}{\partial x}$ around $u^{\left(0\right)}(x,t)$, we obtain its linear form as:

(3.2) $$u(x,t)\frac{\partial u}{\partial x}≊u^{\left(0\right)}(x,t)\frac{\partial u^{\left(0\right)}}{\partial x}+(u^{\left(1\right)}(x,t)-u^{\left(0\right)}(x,t))\frac{\partial u^{\left(0\right)}}{\partial x}\;\;+(\frac{\partial u^{\left(1\right)}}{\partial x}-\frac{\partial u^{\left(0\right)}}{\partial x})u^{\left(0\right)}(x,t).$$

Considering $u^{\left(1\right)}(x,t)≅u(x,t)$, and substituting Eq. (3.2) into Eq. (1.1) gives:

(3.3) $$\frac{\partial u(x,t)}{\partial t}-\varepsilon \frac{\partial^{2}u(x,t)}{\partial x^{2}}+a(x,t)\frac{\partial u(x,t)}{\partial x}+b(x,t)u(x,t)=f(x,t),\forall (x,t)\in \Omega ,$$

where $a(x,t)=u^{\left(0\right)}(x,t),\;\;b(x,t)=\frac{\partial u^{\left(0\right)}(x,t)}{\partial x}$, and $f(x,t)=u^{\left(0\right)}(x,t)\frac{\partial u^{\left(0\right)}(x,t)}{\partial x}$.

To discretize the solution domain, let *M* and *N* be positive integers, then customize the rectangular grid $\Omega_{h}^{k}$ whose nodes are $\left(x_{m},t_{n}\right)$ for m=0,1,2,⋯,M and n=0,1,2,⋯,N. Here $0=x_{0}<x_{1}<\cdots <x_{M}=1,0=t_{0}<t_{1}<\cdots <x_{N}=1$ and $t_{n}=nk,k=\frac{1}{N}$, such that the equidistant grids are considered as

(3.4) $$\left\{ \begin{array}{cc} & x_{m}=mh,\;\;h=\frac{1}{M},\\ & t_{n}=nk,\;\;k=\frac{T}{N}. \end{array}\right.$$

Now, let denote the approximate solution $u_{m}^{n}≅u(x,t)$ at an arbitrary point $\left(x_{m},t_{n}\right)$ of Eq. (3.4) and assume the Eq. (3.3) is satisfied at the point $\left(x_{m},t_{n+0.5}\right)$, that can be written as:

(3.5) $$\frac{\partial u_{m}^{n+\frac{1}{2}}}{\partial t}-\varepsilon \frac{\partial^{2}u_{m}^{n+\frac{1}{2}}}{\partial x^{2}}+a_{m}^{n+\frac{1}{2}}\frac{\partial u_{m}^{n+\frac{1}{2}}}{\partial x}+b_{m}^{n+\frac{1}{2}}u_{m}^{n+\frac{1}{2}}=f_{m}^{n+\frac{1}{2}},\forall (x_{m},t_{n+0.5})\in \Omega_{h}^{k}.$$

For the derivatives with respect to *t*, Taylor series expansion yields

(3.6) $$u_{m}^{n+1}=u_{m}^{n+\frac{1}{2}}+\frac{k}{2}\frac{\partial u_{m}^{n+\frac{1}{2}}}{\partial t}+\frac{k^{2}}{8}\frac{\partial^{2}u_{m}^{n+\frac{1}{2}}}{\partial t^{2}}+\frac{k^{3}}{48}\frac{\partial^{3}u_{m}^{n+\frac{1}{2}}}{\partial t^{3}}+O(k^{4}),$$

(3.7) $$u_{m}^{n}=u_{m}^{n+\frac{1}{2}}-\frac{k}{2}\frac{\partial u_{m}^{n+\frac{1}{2}}}{\partial t}+\frac{k^{2}}{8}\frac{\partial^{2}u_{m}^{n+\frac{1}{2}}}{\partial t^{2}}-\frac{k^{3}}{48}\frac{\partial^{3}u_{m}^{n+\frac{1}{2}}}{\partial t^{3}}+O(k^{4}).$$

Subtracting Eq. (3.7) from Eq. (3.6), yields:

(3.8) $$\frac{\partial u_{m}^{n+\frac{1}{2}}}{\partial t}=\frac{u_{m}^{n+1}-u_{m}^{n}}{k}+TE,$$

where the truncation error

$$TE=-\frac{k^{2}}{24}\frac{\partial^{3}u_{m}^{n+\frac{1}{2}}}{\partial t^{3}}.$$

Taking all the other terms in Eq. (3.5) related to the points (m,n) and (m,n+1), averagely as

$$-\varepsilon \frac{\partial^{2}u_{m}^{n+\frac{1}{2}}}{\partial x^{2}}+a_{m}^{n+\frac{1}{2}}\frac{\partial u_{m}^{n+\frac{1}{2}}}{\partial x}+b_{m}^{n+\frac{1}{2}}u_{m}^{n+\frac{1}{2}}-f_{m}^{n+\frac{1}{2}}=$$

(3.9) $$\frac{1}{2}\{-\varepsilon \frac{\partial^{2}u_{m}^{n+1}}{\partial x^{2}}+a_{m}^{n+1}\frac{\partial u_{m}^{n+1}}{\partial x}+b_{m}^{n+1}u_{m}^{n+1}-f_{m}^{n+1}\;\;-\varepsilon \frac{\partial^{2}u_{m}^{n}}{\partial x^{2}}+a_{m}^{n}\frac{\partial u_{m}^{n}}{\partial x}+b_{m}^{n}u_{m}^{n}-f_{m}^{n}\}.$$

Substituting Eq. (3.8) and Eq. (3.9) into Eq. (3.5), gives the scheme:

(3.10) $$E_{m}^{n+1}u_{m-1}^{n+1}+F_{m}^{n+1}u_{m}^{n+1}+G_{m}^{n+1}u_{m+1}^{n+1}=H_{m}^{n+1},$$

where

$$E_{m}^{n+1}=\frac{-\varepsilon }{h^{2}}-\frac{a_{m}^{n+1}}{2h},\;\;\;F_{m}^{n+1}=\frac{2\varepsilon }{h^{2}}+b_{m}^{n+1}+\frac{2}{k},\;\;\;G_{m}^{n+1}=\frac{-\varepsilon }{h^{2}}+\frac{a_{m}^{n+1}}{2h},$$

and $H_{m}^{n+1}=f_{m}^{n+1}+f_{m}^{n}+\varepsilon \frac{u_{m+1}^{n}-2u_{m}^{n}+u_{m-1}^{n}}{h^{2}}+a_{m}^{n}\frac{u_{m+1}^{n}-u_{m-1}^{n}}{2h}-\left(b_{m}^{n}+\frac{2}{k}\right)u_{m}^{n}$.

Note that, Eq. (3.10) is diagonally dominant due to the conditions $|E_{m}^{n+1}|>0,\;\;|F_{m}^{n+1}|>0,\;\;|G_{m}^{n+1}|>0$ and $\left|F_{m}^{n+1}|>\;\;|E_{m}^{n+1}+G_{m}^{n+1}\right|$ at each ${\left(n+1\right)}^{th}$ level.

## 4 Stability of the method

As different researchers in [19], [12], [3] used, the von-Neumann is applied to investigate the stability of the developed scheme in Eq. (3.10). Thus, assuming that the solution of (3.10) at the grid points $\left(x_{m},t_{n}\right)$ is given by

(4.1) $$u_{m}^{n}=\xi^{n}e^{im\theta },$$

where $i=\sqrt{-1}$ and *θ* is the real number with *ξ* is the amplitude factor. Considering Eq. (4.1) into the homogeneous part of Eq. (3.10) gives the amplitude factor:

(4.2) $$\xi =\frac{\varepsilon \frac{e^{i\theta }-2+e^{-i\theta }}{h^{2}}+a_{m}^{n}\frac{e^{i\theta }-e^{-i\theta }}{2h}-b_{m}^{n}-\frac{2}{k}}{\varepsilon \frac{e^{i\theta }-2+e^{-i\theta }}{h^{2}}+a_{m}^{n+1}\frac{e^{i\theta }-e^{-i\theta }}{2h}+b_{m}^{n}+\frac{2}{k}}.$$

Hence, |ξ|≤1 that guarantees the scheme given in Eq. (3.10) is unconditionally stable.

## 5 Truncation error and consistency of the method

The truncation error TE(h,k) between the exact solution $u(x_{m},t_{n})$ the approximation $U_{m}^{n}$ at the fixed initial iteration of the linearization techniques is given by

$$T(h,k)=\frac{\partial u_{m}^{n+\frac{1}{2}}}{\partial t}-\varepsilon \frac{\partial^{2}u_{m}^{n+\frac{1}{2}}}{\partial x^{2}}+a_{m}^{n+\frac{1}{2}}\frac{\partial u_{m}^{n+\frac{1}{2}}}{\partial x}-b_{m}^{n+\frac{1}{2}}u_{m}^{n+\frac{1}{2}}\;\;+\varepsilon \frac{U_{m+1}^{n+1}-2U_{m}^{n+1}+U_{m-1}^{n+1}}{h^{2}}+$$

(5.1) $$\{a_{m}^{n+1}\frac{U_{m+1}^{n+1}-U_{m-1}^{n+1}}{2h}+\left(b_{m}^{n+1}+\frac{2}{k}\right)U_{m}^{n+1}-\varepsilon \frac{U_{m+1}^{n}-U_{m}^{n}+U_{m-1}^{n}}{h^{2}}\;\;+a_{m}^{n}\frac{U_{m+1}^{n}-U_{m-1}^{n}}{2h}+\left(b_{m}^{n}-\frac{2}{k}\right)U_{m}^{n}\}.$$

From Taylor's series expansion on $U_{m\pm 1}^{n\pm 1}$, $U_{m\pm 1}^{n}$ and substituting into Eq. (5.1) yields:

(5.2) $$T_{m}^{n+1}=-\frac{h^{2}}{6}\left(a_{m}^{n+1}\frac{\partial^{3}U_{m}^{n+1}}{\partial x^{3}}+a_{m}^{n}\frac{\partial^{3}U_{m}^{n}}{\partial x^{3}}-\frac{\varepsilon }{2}\left(\frac{\partial^{4}U_{m}^{n+1}}{\partial x^{4}}+\frac{\partial^{4}U_{m}^{n}}{\partial x^{4}}\right)\right)\;-\frac{k^{2}}{24}\frac{\partial^{3}U_{m}^{n+\frac{1}{2}}}{\partial t^{3}}+\left(h^{4},k^{4}\right).$$

Hence, this Eq. (5.2) verifies the consistency of the formulated methods as the mesh size parameters (h,k)→(0,0), one can obtain TE(h,k)→(0,0). Thus, consistency is shown in Eq. (5.2) with stability condition provided in Eq. (4.2).

Therefore, Eq. (3.10), is convergent scheme by Lax's equivalence theorem, one can refer to the details in [1], [2], [3], [17], [18], [19], [20], [21]. Further, the local truncation error is bounded with confirmation of its norm as

(5.3) $$||TE(h,k)||\leq C_{1}h^{2}+C_{2}k^{2}≅C(h^{2}+k^{2}),$$

where

$$C_{1}=\frac{1}{6}{\left\|a_{m}^{n+1}\frac{\partial^{3}U_{m}^{n+1}}{\partial x^{3}}+a_{m}^{n}\frac{\partial^{3}U_{m}^{n}}{\partial x^{3}}-\frac{\varepsilon }{2}\{\frac{\partial^{4}U_{m}^{n+1}}{\partial x^{4}}+\frac{\partial^{4}U_{m}^{n}}{\partial x^{4}}\}\right\|}_{\infty },C_{2}=\frac{1}{24}{\left\|\frac{\partial^{3}U_{m}^{n+\frac{1}{2}}}{\partial x^{3}}\right\|}_{\infty }.$$

Remark Let u(x,t) be the solution of the problem in Eq. (3.3), and $U_{m}^{n}$ be the approximate solution of the fully discrete scheme given in Eq. (3.10). Then as indicated in Eq. (5.3), the error estimate is given by

$$|u(x,t)-U_{m}^{n}|\leq C(h^{2}+k^{2}),\;\;\;0\leq m\leq M,\;\;0\leq n\leq N.$$

Therefore, the formulated method is a consistent and stable scheme that implies convergent scheme by Lax's equivalence, [6], [25], [26].

## 6 Numerical illustrations and discussions

In this section, two numerical examples have been considered. Their exact solutions are not available, so that the errors are evaluated by using the double mesh principle [1], [5], [14], [20], [24] given by:

$$E_{\varepsilon }^{M,N}=\max_{(x_{m},t_{n})\in {\bar{\Omega }}_{M}^{N}}|U_{m}^{n}-U_{2m}^{2n}|,$$

where $U_{m}^{n}$ and $U_{2m}^{2n}$ are the approximate solution on the fully discritized domain. From these values, we determine the corresponding rate of convergence by the formula

$$P_{\varepsilon }^{M,N}=\frac{logE_{\varepsilon }^{M,N}-logE_{\varepsilon }^{2M,2N}}{log2}.$$

The computed maximum absolute errors and rate of convergence are given in Tables, and further simulations are in terms of Figures provided.

Example 6.1 Consider the singularly perturbed Burgers' equation:

$$\left\{ \begin{array}{cc} & \frac{\partial u(x,t)}{\partial t}-\varepsilon \frac{\partial^{2}u(x,t)}{\partial x^{2}}+u(x,t)\frac{\partial u(x,t)}{\partial x}=0,\;\;\forall (x,t)\in (0,1)\times (0,1],\\ & u(x,0)=sin\pi x,\;\;\;0\leq \;x\;\leq 1,\\ & u(0,t)=0,\;\;u(1,t)=0,\;\;\;0<\;t\;\leq 1. \end{array}\right.$$

Example 6.2 Consider the singularly perturbed Burgers' equation:

$$\left\{ \begin{array}{cc} & \frac{\partial u(x,t)}{\partial t}-\varepsilon \frac{\partial^{2}u(x,t)}{\partial x^{2}}+u(x,t)\frac{\partial u(x,t)}{\partial x}=0,\;\;\forall (x,t)\in (0,1)\times (0,1],\\ & u(x,0)=x(1-x^{2}),\;\;\;0\leq \;x\;\leq 1,\\ & u(0,t)=0,\;\;u(1,t)=0,\;\;\;0<\;t\;\leq 1. \end{array}\right.$$

Under Table 1, Table 2, Table 4, we provide the maximum absolute errors to validate the efficiency of the scheme. Further, Table 3 confirms that the formulated method is second-order convergent.

**Table 1:** Computed and comparison of maximum absolute errors for Example 6.1.

*ε*↓*M*/*N*→	64/20	128/40	256/80	512/160
Present–Method				
10^−2^	2.0667e − 03	5.4043e − 04	1.3664e − 04	3.4288e − 05
10^−4^	2.2265e − 03	5.8421e − 04	1.4836e − 04	3.7200e − 05
10^−6^	2.2281e − 03	5.8482e − 04	1.4849e − 04	3.7232e − 05
Result in [20]				
10^−2^	1.1303e − 01	6.1846e − 02	2.7592e − 02	1.4072e − 02
10^−4^	2.5946e − 01	1.4418e − 01	6.8426e − 02	3.2474e − 02
10^−6^	2.7194e − 01	1.5667e − 01	6.9863e − 02	3.3902e − 02

**Table 2:** Computed maximum absolute errors for Example 6.1.

*ε*↓*M* = *N*→	16	32	64	128	256	512
10^−2^	3.5203e − 03	9.4233e − 04	2.4054e − 04	6.0423e − 05	1.5130e − 05	3.7838e − 06
10^−4^	4.1843e − 03	1.1071e − 03	2.8300e − 04	7.1034e − 05	1.7776e − 05	4.4452e − 06
10^−6^	4.2047e − 03	1.1124e − 03	2.8450e − 04	7.1411e − 05	1.7871e − 05	4.4688e − 06
10^−8^	4.2050e − 03	1.1125e − 03	2.8452e − 04	7.1416e − 05	1.7872e − 05	4.4691e − 06
10^−10^	4.2050e − 03	1.1125e − 03	2.8452e − 04	7.1416e − 05	1.7872e − 05	4.4691e − 06
10^−12^	4.2050e − 03	1.1125e − 03	2.8452e − 04	7.1416e − 05	1.7872e − 05	4.4691e − 06
10^−14^	4.2050e − 03	1.1125e − 03	2.8452e − 04	7.1416e − 05	1.7872e − 05	4.4691e − 06
10^−16^	4.2050e − 03	1.1125e − 03	2.8452e − 04	7.1416e − 05	1.7872e − 05	4.4691e − 06

**Table 3:** Computed rate of convergence for Example 6.1.

*ε*↓*M* = *N*→	16	32	64	128	256
10^−2^	1.9014	1.9700	1.9931	1.9977	1.9995
10^−4^	1.9182	1.9679	1.9942	1.99869	1.9996
10^−6^	1.9183	1.9672	1.9942	1.9985	1.9997
10^−8^	1.9183	1.9672	1.9942	1.9985	1.9996
10^−10^	1.9183	1.9672	1.9942	1.9985	1.9996
10^−12^	1.9183	1.9672	1.9942	1.9985	1.9996
10^−14^	1.9183	1.9672	1.9942	1.9985	1.9996
10^−16^	1.9183	1.9672	1.9942	1.9985	1.9996

**Table 4:** Comparison of maximum absolute errors for Example 6.2.

*ε*↓*M*/*N*→	32/20	64/40	128/80	256/160	512/320
Present Method					
2^−10^	2.5222e − 04	6.7547e − 05	1.7077e − 05	4.2892e − 06	1.0730e − 06
2^−12^	2.5527e − 04	6.8045e − 05	1.7231e − 05	4.3246e − 06	1.0818e − 06
2^−14^	2.5603e − 04	6.8169e − 05	1.7270e − 05	4.3334e − 06	1.0842e − 06
2^−16^	2.5622e − 04	6.8201e − 05	1.7280e − 05	4.3356e − 06	1.0847e − 06
Result in [1]					
2^−10^	8.3339e − 02	6.6120e − 02	4.0769e − 02	1.9284e − 02	9.1775e − 03
2^−12^	1.8762e − 01	8.4106e − 02	5.7234e − 02	3.8309e − 02	1.9041e − 02
2^−14^	1.9755e − 01	1.5347e − 01	8.3864e − 02	4.6945e − 02	2.5508e − 02
2^−16^	2.1340e − 01	1.5016e − 01	1.1582e − 01	6.1958e − 02	3.3441e − 02

**Figure 1:**
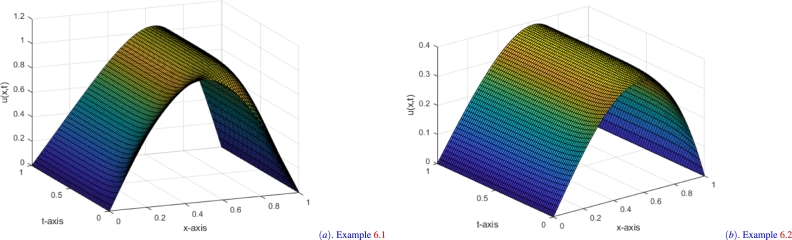
Solution profiles for the Examples when *M* = *N* = 64 and *ε* = 10^−4^.

**Figure 2:**
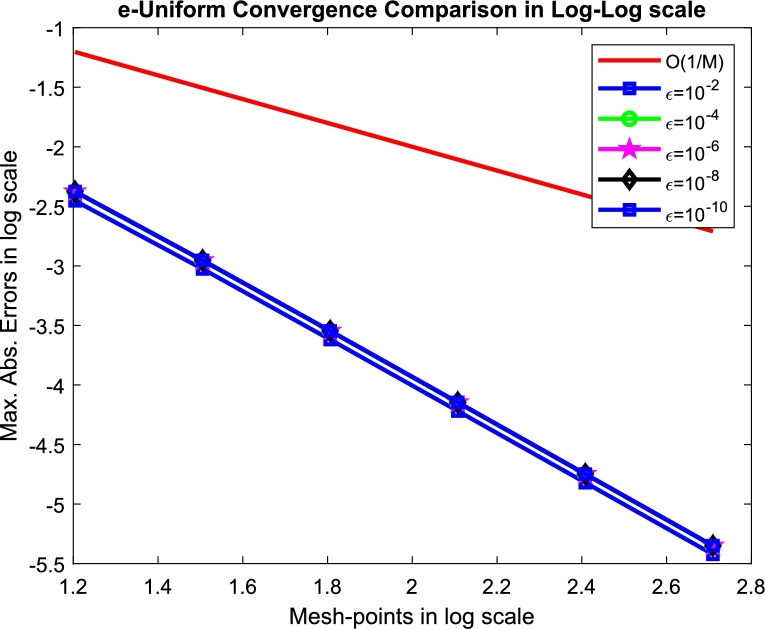
Log-log plots for Example 6.2.

Furthermore, Table 3 illustrates the second-order convergence with robustness of the present method. Fig. 1 shows the solution profile for Example 6.1, and the log-log plot (Fig. 2) illustrated for Example 6.2.

## 7 Conclusion

Now, a second-order robust scheme is presented for solving singularly perturbed Burgers' equation. To develop this scheme, we use standard second-order finite difference approximations for the two independent variables. Also, the obtained experimental results as indicated in Tables confirm the betterment of the obtained result than some existing one in the literature.

## Declarations

### Author contribution statement

Masho Jima Kabeto: Conceived and designed the experiments; Performed the experiments; Analyzed and interpreted the data.

Gemechis File Duressa: Analyzed and interpreted the data; Wrote the paper.

### Funding statement

This research did not receive any specific grant from funding agencies in the public, commercial, or not-for-profit sectors.

### Data availability statement

Data included in article/supplementary material/referenced in article.

### Declaration of interests statement

The authors declare no conflict of interest.

### Additional information

No additional information is available for this paper.
